# Combination of unsupervised discretization methods for credit risk

**DOI:** 10.1371/journal.pone.0289130

**Published:** 2023-11-27

**Authors:** José G. Fuentes Cabrera, Hugo A. Pérez Vicente, Sebastián Maldonado, Jonás Velasco

**Affiliations:** 1 Departamento de Ingeniería Química, Industrial y de Alimentos, Universidad Iberoamericana Ciudad de Mexico, Mexico City, México; 2 Facultad de Estudios Superiores Acatlán, UNAM, Naucalpan de Juárez, México; 3 Department of Management Control and Information Systems, School of Economics and Business, University of Chile, Santiago, Chile; 4 Instituto Sistemas Complejos de Ingeniería (ISCI), Santiago, Chile; 5 CONAHCYT-Centro de Investigación en Matemáticas (CIMAT), Guanajuato, México; National University of Computer and Emerging Sciences, PAKISTAN

## Abstract

Creating robust and explainable statistical learning models is essential in credit risk management. For this purpose, equally spaced or frequent discretization is the de facto choice when building predictive models. The methods above have limitations, given that when the discretization procedure is constrained, the underlying patterns are lost. This study introduces an innovative approach by combining traditional discretization techniques with clustering-based discretization, specifically *k* means and Gaussian mixture models. The study proposes two combinations: Discrete Competitive Combination (DCC) and Discrete Exhaustive Combination (DEC). Discrete Competitive Combination selects features based on the discretization method that performs better on each feature, whereas Discrete Exhaustive Combination includes every discretization method to complement the information not captured by each technique. The proposed combinations were tested on 11 different credit risk datasets by fitting a logistic regression model using the weight of evidence transformation over the training partition and contrasted over the validation partition. The experimental findings showed that both combinations similarly outperform individual methods for the logistic regression without compromising the computational efficiency. More importantly, the proposed method is a feasible and competitive alternative to conventional methods without reducing explainability.

## 1 Introduction

Credit risk is one of the most popular fields of application of machine learning techniques due to the abundance of datasets and the number of developments deployed in the financial sector and in particular in retail credit [1, 2].

When a machine learning classification problem is faced, it can be mistakenly assumed that the problem is solved due to the large number of efforts made by the scientific community to produce multiple theoretical solutions. However, the complexity of the problem increases as applications demand more sophisticated methods to meet the specific requirements of the niche where they are encountered [3–5].

To this end, the methodology for knowledge discovery in large data volumes CRISPDM (Cross Industry Standard Process for Data Mining) [6] with the subsequent steps to follow: business understanding, data understanding, data preparation, modeling, assessment, and deployment, is widely used in the field. As mentioned earlier, each step features large samples of research work, seeking efficiencies in these crucial stages necessary to resolve the application problem satisfactorily [7–9].

Despite the previous, a lack of focus on the explainability methods through generalized linear models is present in practice. Also, simple discretization methods based on equal frequency or distance are still used as the first option.

It is essential to notice that logistic regression, in combination with discretization methods, is the current gold standard for credit scoring, which is the main application for this study. This is due to regulatory constraints (Basel II/III), which prohibit the use of black box models such as neural networks [10].

Although decision trees (DTs) are white box models and regulatory restrictions do not apply, they do not benefit from a discretization step since DTs aim is to generate an optimal binning to define if/then rules that maximize the discriminative power of the variables. Therefore, a previous discretization step would be redundant and negatively affect the method’s performance.

On the other hand, artificial neural networks (ANNs) are designed to capture complex relationships between (numerical) variables. Using binning as a preprocessing step limits the ability of ANNs to construct non-linear functions, affecting the predictive performance negatively. Additionally, improving the interpretability of an ANN with a discretization step is not possible.

Discretization of continuous predictors is a widely used technique for data preprocessing [11–13], which is a crucial stage prior to entering data in the learning process in multiple machine learning classifiers. The above is based on the fact that such preprocessing captures patterns and minimizes statistical noise by grouping similar elements and facilitating the mapping between the feature space X and the binary response vector $\vec{y}$.

Several approaches of discretization methods are used to pursue different objectives, such as monotonic risk clusters [13], optimal clusters regarding the response feature [11, 14], or clustering to improve a specific classifier [15, 16]. However, little attention is given to combination methods.

Combining different discretization approaches can overcome the information intrinsically lost during the clustering process without lack of explainability of the resulting models, which is of utmost importance in the financial and healthcare industry [17–20].

The proposal of this paper outlines two innovative approaches. Firstly, to assemble the unsupervised discretization methods most commonly used in the industry: equal distance and equal frequency methods to improve the predictions in a classification problem using a logistic regression model. Secondly, to add two clustering algorithms (*k* means and Gaussian mixture models) to the combination to produce more similar and homogeneous segments. Multiple supplementary segments can be incorporated into the model by using this approach, improving the classification quality of the classifier without losing interpretability.

The paper is articulated as follows: in Section 2, the necessary theoretical background to read this paper is briefly reviewed. In Section 3, the available related papers in the literature are discussed. Section 4 discusses the details on the availability of the software, data description, data processing and experimentation derived from this paper. In Section 5, the proposed combination algorithm approach is described, as well as the two implementation variations raised: Discrete Competitive Combination (DCC) and Discrete Exhaustive Combination (DEC). Additionally, the experimentation methodology is underlined. Experimental results and their corresponding discussion are presented in Section 6. Finally, the conclusions and possible future lines of work are addressed in Section 7.

## 2 Methodological background

This section will briefly describe the critical and necessary theoretical concepts used in this research paper.

### 2.1 Discretization methods

#### 2.1.1 Equal distance method

A simple discretization method in which the continuous feature will be divided into equal intervals [13]. The number of intervals must be determined *a priori*. With this understanding, let *A* be a feature with domain *D*_*A*_ ∈ (*a*, *b*) for a>b,a,b∈R and *k* the number of intervals of the discretization. Then, the boundaries of each interval will be:

$$\left\{a+i\frac{\left(b-a\right)}{k}\;|\;i=0,\ldots ,k\right\}$$

#### 2.1.2 Equal frequency method

This method also requires establishing the number of intervals in which feature *A* will be divided. The equality of intervals will not be set through distances but through the frequency associated with each interval [13]. For this, the data domain’s percentile calculation (denoted *p*). Therefore, given *D*_*A*_ ∈ (*a*, *b*) for a>b,a,b∈R and *k* the number of desired intervals, then the interval boundaries will be provided by:

$$\left\{a\right\}\cup \{p_{\frac{100i}{k}}\;|\;i=1,\ldots ,k-1\}\cup \left\{b\right\}$$

#### 2.1.3 *k* means discretization method

In the case of *k* means [21], the discrete intervals will be established by unsupervised learning in which the space will only be formed by feature *A*. The method works through the minimization of the cost of the clusters formed. Then, given a set of points: *C*_*j*_ = {*i* ∈ {1, …, *n*}|*z*^(*j*)^} represents *x*^(*j*)^, then:

$$\text{Cost}(z^{\left(1\right)},\ldots ,z^{\left(k\right)})=\sum_{i=1}^{n}\underset{j=1,\ldots ,k}{\text{min}}{\left\|x^{\left(i\right)}-z^{\left(j\right)}\right\|}^{2}$$

For this purpose, the *k* means algorithm finds the best clusters for the centroids and the best centroids for the clusters. Iterations are performed according to the following:

1. Initialize the centroids *z*^(1)^, …, *z*^(*k*)^
2. Repeat until there is no significant change in the Cost function:
  (a) For each *j* = 1, …, *k*:
    *C*_*j*_ = {*i*|*x*^(*i*)^ is the closest to *z*^(*j*)^}
  (b) For each *j* = 1, …, *k*:

$\;z^{\left(j\right)}=\frac{1}{|C_{j}|}\sum_{i\in C_{j}}x^{\left(i\right)}$

#### 2.1.4 Gaussian mixture model discretization method

Gaussian mixture models [22] is an unsupervised learning method that produces *k* clusters. The assignment of each cluster is produced according to the probabilities produced by a Gaussian distribution. The one with the highest probability will be selected as the representative cluster. The Gaussian mixture model is a weighted sum of multivariate normal distributions as the following:

$$\sum_{j=1}^{k}p_{j}N(x;\mu^{\left(j\right)},\sigma_{j}^{2}I)$$

where *p*_*j*_ represents the weights of the mixture, *μ*^(*j*)^ the mean vector, and $\sigma_{j}^{2}I$ the covariance matrix.

### 2.2 Classification metrics

#### 2.2.1 Area under the ROC curve (AUC)

As indicated in [23], the procedure for obtaining AUC is based on the confusion matrix resulting from training with the peculiarity that the probability cutoff to determine the class is measured in different *k* points, thus producing the two-dimensional coordinates for the formation of a curve called receiver operating characteristic or ROC curve. Each coordinate corresponds to the ordered pair of sensitivity and 1 minus specificity. Subsequently, the indicator will be obtained from the calculation of the area under the mentioned curve, where the reference value 0.5 indicates an identical classification to the random effect and 1.0 indicates a perfect classification. Therefore, values closer to 1.0 indicate a model with better classification.

#### 2.2.2 Kolmogorov Smirnov

The statistic of the Kolmogorov Smirnov (KS) test is based on the maximum difference between the cumulative distributions of an empirical and a theoretical distribution [24] (The positive and negative classification will take such roles indistinctively but not simultaneously). Higher values of KS value indicate better classification, being zero, its minimum value in the presence of identical distributions.

### 2.3 Logistic regression

Logistic regression allows modeling a dichotomous outcome in a probabilistic manner where *p* represents the probability of occurrence of an event in particular. The complement of *p* is denoted by *q* = 1 − *p*. The modeled probability approach will be based on the odds ratio, calculated using the expression:

$$\frac{p}{q}=\frac{p}{1-p}$$

If each independent variable *x*_*i*_ proportionally contributes to the response feature, the weighted sum of the predictors in linear correspondence with the logarithm of the odds ratio is proposed, hence:

$$\text{ln}(\frac{p}{1-p})=\beta_{0}+\sum_{i=1}^{n}\beta_{i}x_{i}$$

Generating an expression for *p*:

$$\begin{array}{c} p=\frac{1}{1+\text{exp}\left(-\beta_{0}-\sum_{i=1}^{n}\beta_{i}x_{i}\right)} \end{array}$$ (1)

The previous expression is a logistic function (sigmoid), which returns values between 0 and 1. These will be interpreted as the probability of occurrence of the event for each vector presented. *β*_*i*_ parameters are usually estimated using the maximum likelihood method [25].

### 2.4 Weight of Evidence and information value

The *Weight of Evidence* (WoE) encoding converts the risk associated with the probability of occurrence of an outcome in a linear scale which is easily interpreted [10]. To generate such transformation, the following is taken into consideration:

Let *P* be the number of occurrences of a certain outcome in particular and *N* the number of non-occurrences. Then, the WoE encoding for the *i*-th attribute associated with a particular feature is given by:

$${\text{WoE}}_{i}=\text{log}(\frac{N_{i}}{\sum_{i=1}^{n}N_{i}}/\frac{P_{i}}{\sum_{i=1}^{n}P_{i}})$$

WoE encoding is suitable for logistic regression because a linear correspondence with the sigmoid function is present in its structure. A consequence of the WoE transformation consists in the capability to measure the classification quality of each explanatory variable through the statistic known as *Information Value* (IV), which is calculated using the equation:

$$\text{IV}=\sum_{i=1}^{n}[(\frac{N_{i}}{\sum_{i=1}^{n}N_{i}}-\frac{P_{i}}{\sum_{i=1}^{n}P_{i}}){\text{WoE}}_{i}]$$

Information Value (IV) corresponds to the Kullback-Leibler divergence between the event, and non-event distributions [26].

## 3 Related work

Credit risk is one of the field applications in which the aim is to propose a simple and interpretable model to find the pattern that will determine if a borrower will or will not repay the granted loan [27]. Ai et al.’s [28] work presents an approach through the order of categorical data to cluster information in the face of the potential for fraud in claims under the insurance policies industry, the method used by the author is called RIDIT (Relative to an Identified Distribution Integral Transformation), in which an ordinal measurement is assigned to discrete variables, in this regard, the idea is compatible with the proposal of this paper. On the other hand, there are several applications, such as those shown by Nehrebecka [29] Melchiori [30], and Bunker [31] related to the default risk of companies, change of category of regulatory classification and improvement of the classification quality by adding information, respectively. In particular, both in [29, 30], there are regulatory implications for having explainable methods, such as the one raised in this paper, which is essential. Moreover, regarding the effects of the systemic impact of the COVID-19 pandemic for measuring the effect in the probability of default for non-financial companies using a stress-testing framework is proposed by Nehrebecka [32].

Additionally, the applications shown by Wang [33] and Zhang [34] outline approaches based on deep learning for peer-to-peer lending. In Wang’s case, an approach based on long-short term memory (LSTM) architectures is used, whereas, in Zhang’s paper, deep networks and gradient boosting models are assembled to produce the online integrated credit scoring model (OICSM), adding online update capabilities. Furthermore, Guo’s paper [35] proposes a self-adaptive method to assemble classifiers applied to credit risk. The method involves creating base classifiers and selecting the optimal combination through Bayesian optimization. Finally, some of the datasets used in this paper [36–38] were also selected by Guo for experimentation.

Many techniques with different approaches, such as segment restrictions and desirable engineering characteristics regarding discretization methods, are widely present in the literature. An interesting summary of the taxonomy of such methods can be found in [12]. However, in this review, no references were found regarding a discretization proposal based on Gaussian mixture models as proposed in this paper. Moreover, it should be noted that discretization based on clustering is implemented in the scikit learn [39] machine learning library through the *k* means method, as well as in different CRAN repository packages in the R programming language [40] due to which there is a history of widespread use of a similar approach.

A broad range of approaches with similar aims to the proposal of this paper can be found in connection with discretization methods. For example, Adeodato [11] proposes a method to maximize the KS (Kolmogorov Smirnov) statistic through clustering in the discretization process. In addition, in Cano’s [41] paper, a method called LAIM (Label Attribute Interdependence Maximization) is proposed to have a specific discretization for multinomial classification problems.

A comparison between discretization methods is discussed in Lund’s [15] paper regarding the added value of discretization through the WoE (Weight of Evidence) transformation versus an approximation based on the binarization of the segments of the discrete predictors. In Saia’s [42] paper, discretization alternatives are proposed to improve the classification quality applied to credit scoring through the method called Discretized Enriched Data (DED).

The method mentioned above consists of two steps: the first is to reduce the patterns of predictors through discretization, and the second is to enrich data through meta predictors. Finally, Navas [14] proposes the formation of optimal discretization groups through mathematical programming.

Following the aim of this paper’s research (to discretize and combine to improve classification quality applied to credit risk), alternative studies that supplement this effort are present. For instance, Wojciak [43] compares four discretization methods to assemble them into a Bayesian network. On the other hand, in Yang’s [16] research, discretization methods are combined into a Naive Bayes classifier. In Hsieh’s [44] work, an assembly of several methods is generated to boost the classifier applied to credit scoring. Finally, Vejkanchana [45] proposes an innovative way of generating groups in the discretization process through supervised modeling using evolutionary computation, allowing the user to determine the form and number of desired clusters through a constrained optimization.

As far as Raymaeker’s [46] investigation is concerned, a fascinating approach can be found based on the creation of non linear segments through splines, seeking to ensure the capture of complex patterns within data without detriment to interpretability. The latter has similarities with this paper by aligning in vision and differing through the approach of discrete segment formation. No less important is to stress that papers such as Silva’s [17], Raymaekers’s [47] and Iftikhar’s [48, 49] highlight the importance of the resulting classifiers’ explainability in multiple industries and cases of use, which is one of the purposes of this paper.

The main contributions of this research are focused on supplementing the notorious efforts of the scientific community to provide suitable tools for their practical application; for this reason, the proposal of this paper focuses on supplementing and joining the efforts discussed regarding discretization techniques, improvement of classification quality in a classifier and its direct applications in credit risk.

## 4 Data processing and software availability

The software created during this research is available in the GitHub repository https://github.com/JGFuentesC/creditrisk_discretization_combination. The repository contains everything necessary to process raw data, replicate the computational experiment and delve into the programming logic used to develop the proposed methodology.

### 4.1 Data description

A computational experiment is proposed based on proving the competitiveness of the combination in various datasets. The chosen datasets have helpful features to achieve the aim of this study, namely: credit risk classification for several retail and business banking products, sufficient data volume, presence of continuous variables, and presence in different related works. Following is a list of the used datasets in detail:

1. Australian credit—Australian [37]
2. Microfinance loan credit scoring—Farmers [50]
3. German Credit—German [36]
4. Give me some credit—Give-me-some-credit [51]
5. HMEQ—Hmeq [52]
6. Japan credit—Japan [38]
7. Lending club—Lending-club [53]
8. Mexican credit (used in a previous paper of the authors)—Mexico [27]
9. Mortgage—Mortage [52]
10. Polish companies—Polish [54]
11. Taiwan credit—Taiwan [55]

The basic metadata of each dataset is shown in Table 1. Note that when a dataset surpasses 10,000 observations, a simple random sampling of 5,000 (for maintaining the original event distribution) is taken for conducting the experiment.

**Table 1:** Dataset description.

Dataset	Num. Obs.	Predictors	Pos. Cases	Neg. Cases
Australian	690	6	307	383
Farmers	5000	12	2164	2836
German	959	7	275	684
Give-me-some-credit	5000	10	327	4673
Hmeq	5960	10	1189	4771
Japan	690	4	383	307
Lending-club	5000	77	693	4307
Mexico	5000	14	579	4421
Mortgage	5000	15	112	4888
Polish	5000	64	239	4761
Taiwan	5000	6	534	4466

Dataset basic description.

### 4.2 Dataset topology

**Fig 1:**
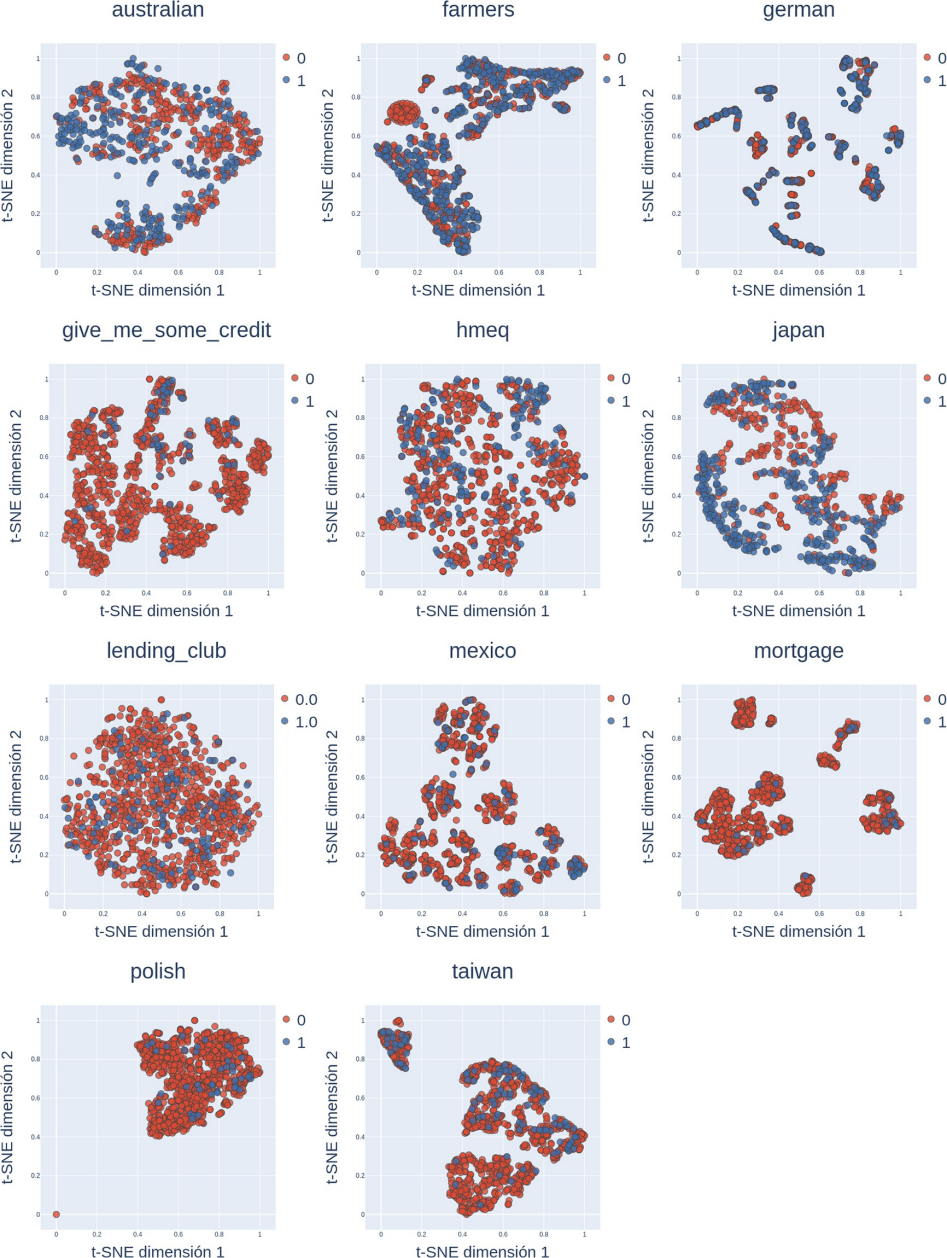
Datasets topology. Data topology, t-SNE with event based hue.

To illustrate the variety of the chosen data, two dimensions data topology in each one of the datasets that will be studied is shown in Fig 1. The objective of the display is to graphically show the variability in the datasets through a standard of comparison. The information presented is obtained using the technique known as *t* Distributed Stochastic Neighbor Embedding (t-SNE) [56], which represents the predictor hyperspace in its most likely form through probability distribution fitting in pairs of objects in the high dimensional space in order to ensure that similar objects are assigned to higher probabilities. Subsequently, the method carries out a similar process in a low dimensional space to minimize the Kullback-Leibler divergence between both distributions.

With respect to data topology shown in Fig 1, the following characteristics are highlighted:

- Australian, Farmers, and Japan are the only datasets showing class balance.
- Complex patterns are likely to emerge in datasets closest to real-world conditions such as lending club, mexico, polish, and mortgage and give me some credit.
- In general, no linear separation is shown.

As can be observed, the presented datasets have a broad range of topologies. Therefore, the preceding indicates the soundness of this paper’s proposal, whereby the generalization capability for multiple configurations and not only through classic or linearly separable examples are tested.

## 5 Methodology

### 5.1 Combination method definition

This paper proposes a combination method that considers the information contributed by each discretization method to generate the most accurate classifier without loss of interpretability. Moreover, the information captured by a particular discretization method will not necessarily consider the benefits of its counterparts. This principle is used similarly in the algorithms based on decision trees, as seen in Chen’s [57]. In that work, the memory capacity is suppressed under the further inclusion of each previously chosen predictor, except that a sub-partition will improve the classification capacity. The proposal of this paper uses this approach analogously; however, the way to capture additional information will not be through successive partitions of the same variable but through the compliment that a partition determined by a different rule may provide.

**Fig 2:**
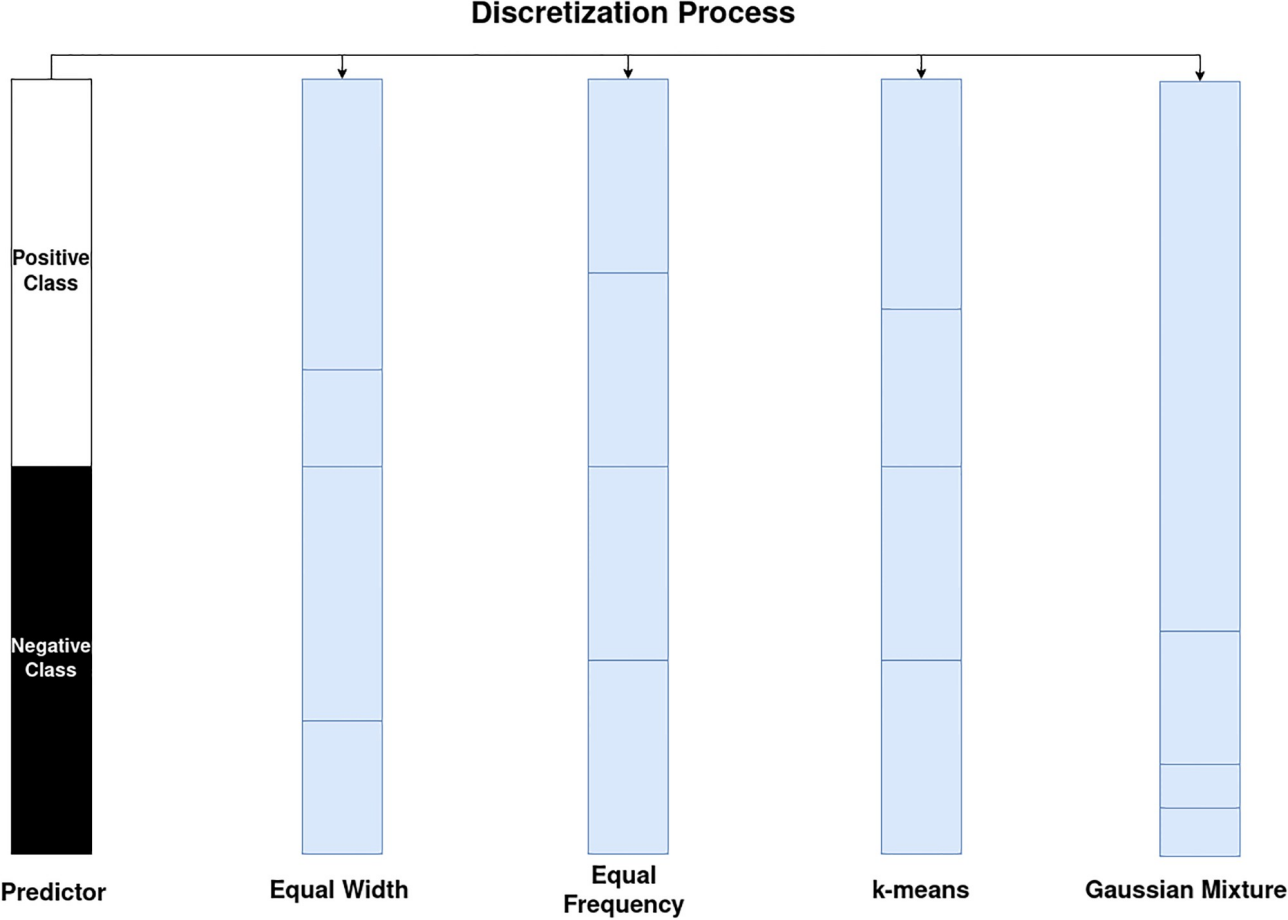
Intuitive discretization process. Intuitive differentiation between segments produced by different discretization techniques.

This approximation is represented intuitively in Fig 2:

Fig 1 shows how different techniques will produce distinct segments in each predictor feature; in addition, the proportion (purity) of the partition between the elements of the positive and negative class of the binary classifier will also be different. Consequently, the selection of the technique that generates a purer partition will be chosen; hence, in contrast to the individual selection, any combination is expected to deliver better results experimentally.

In order to carry out the task, each one of the continuous predictors is discretized in *k* = 2, …, 10 segments according to each method. The choice of the *k* range is based on the fact that being interpretability a critical element of the proposed method, having more than 10 segments would make interpretation difficult.

In this manner, if *n* is the number of predictors, a total of $\sum_{k=2}^{10}4nk=216n$ univariate segments in the predictors’ space will be generated. Therefore, the two variations that may derive from this combination are described below:

- Discrete Competitive Combination (DCC): let *m* be the discretization method used and *k* the number of segments in which each predictive feature *x* will be discretized, *m* and *k* are chosen in such a manner that:
  $$\underset{m,k}{\text{arg}\;\text{max}}\;\text{IV}\left(x,m,k\right),\;\forall \;x\in X$$
  In this manner, the discretization methods *compete* with each other to determine the method/number combination of segments that maximizes each predictor’s classification quality.
- Discrete Exhaustive Combination (DEC): in this variation, the four discretization methods chosen are included in a comprehensive manner. Let M be the set of discretization methods, *k* is chosen for each predictor x∈X such as:
  $$\underset{k}{\text{arg}\;\text{max}}\;\text{IV}\left(x,m,k\right),\;\forall \;m\in M$$
  The above forces the inclusion of each discretization method to provide the additional information that each method contributes individually to the combination.

After this selection, the generated segments enter as predictive features of the logistic regression (after WoE transformation), thus combining the additional information in each predictor generating a more powerful classifier instead of only using individual methods.

Finally, the logistic regression parameters are adjusted to measure the complete model’s classification quality and enable comparison between methods. The algorithms Discrete Competitive Combination (DCC) and Discrete Exhaustive Combination (DEC) are shown as Algorithm 1 and 2, respectively. Each element of the set of discretization methods M={GMM,KME,EQF,EQD}, corresponds to the Gaussian mixture models, *k* means, equal frequency and equal distance, respectively. X refers to the matrix for continuous predictors. *θ* is the vector of logistic regression parameters. Vector $\vec{y}$ contains the positive and negative labels of the training set.

**Algorithm 1** Discrete Competitive Combination (DCC)

1: **Procedure** DCC(X)

2:  *k* ← 2

3:  **while** *k* ≤ 10 **do**

4:   **for** *m* in M **do**

5:    Discretize per method *m* in *k* segments

6:   *k* ← *k* + 1

7:  Select best *k* for each *m*

8:  Select best *m*

9:  Transform to WoE encoding using the training partition of set $\left(X,\vec{y}\right)$

10:  Estimate $\hat{\theta }|\left(X,\vec{y}\right)$

**Algorithm 2** Discrete Exhaustive Combination (DEC)

1: **procedure** DEC(X)

2:  *k* ← 2

3:  **while** *k* ≤ 10 **do**

4:   **for** *m* in M **do**

5:    Discretize per method *m* in *k* segments

6:   *k* ← *k* + 1

7:  Select best *k* for each *m*

8:  Transform to WoE encoding using the training partition of set $\left(X,\vec{y}\right)$

9:  Estimate $\hat{\theta }|\left(X,\vec{y}\right)$

### 5.2 Proposed methodology

**Fig 3:**
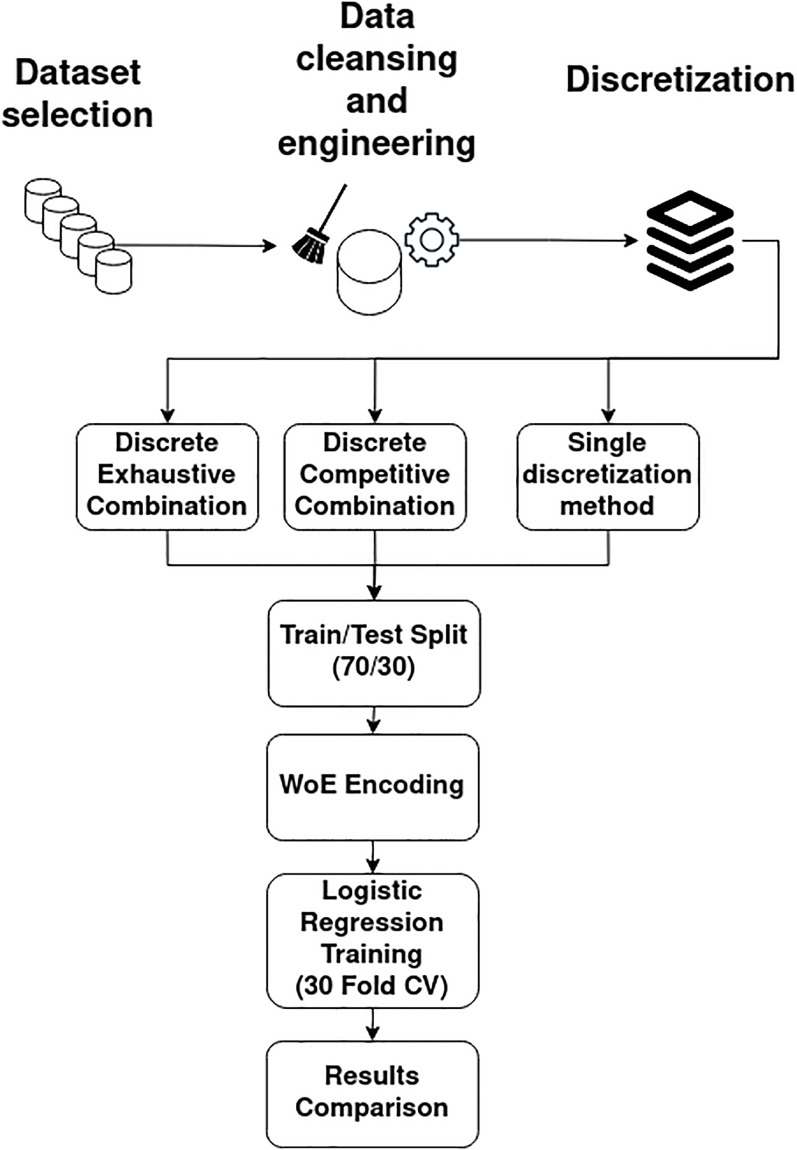
Proposed methodology. Proposed methodology steps illustration diagram.

The methodology used in this paper is illustrated in Fig 3. The proposed methodology allows comparison of the proposed combination’s competitiveness through the systematic application of a standard machine learning process with the addition of pre-processing (discretization) and the innovative predictor selection (combination) algorithm. The steps of this methodology are listed below.

1. Selection of datasets related to credit risk.
2. Data cleansing and engineering: removal of inconsistencies, standardize names of predictors, eliminate categorical variables.
3. Discretization in *k* segments, *k* = 2, 3, …, 10 for each method (Gaussian mixture models, *k* means, equal frequency, and equal distance).
4. Predictor selection.
  (a) Selection of the best method and number of segments for each variable (DCC, Algorithm 1).
  (b) Selection of the best number of segments including a discretization for each method (DEC, Algorithm 2).
  (c) Selection of the best number of segments only using one method at a time.
5. Data partition (70% training and 30% validation). A commonly used ratio in practice [58].
6. WoE encoding.
7. 30-fold cross-validation logistic regression training. Literature suggests that ten folds usually suffice [59]. In order to strengthen the robustness of the experiment, 30 folds were used.
8. Comparison of results.

## 6 Results and discussion

In this section, the steps of the experimental framework used to generate the necessary data for comparing the single discretization methods against the proposed discretization combinations are described below.

1. Combination selecting the best number of segments and method for each variable, the methods compete with each other (DCC, Algorithm 1).
2. Combination selecting the best number of segments for each method and predictor, the inclusion of each discretization method in the combination (DEC, Algorithm 2) is forced.
3. Training using the best number of segments for each variable by the Gaussian mixture model (GMM) discretization method.
4. Training using the best number of segments for each variable by the *k* means (KME) discretization method.
5. Training using the best number of segments for each variable by the Equal frequency (EQF) discretization method.
6. Training using the best number of segments for each variable by the Equal distance (EQD) discretization method.

The main goal in the preceding stages was to evaluate the effectiveness of the proposed combinations in enhancing the predictive capabilities of a binary classifier. To test the proposed combinations, computational experiments were carried out on a system with an 11th Gen Intel Core i7-11800H @ 16x 4.6GHz and 32 GB of RAM, running the Linux operating system with Ubuntu Desktop release 22.04. All computations were performed using Python version 3.8.10. Specific packages and their corresponding versions are detailed in Section 4.

Statistics are computed by taking the validation samples outcome during the 30 cross validation folds for each of the 11 datasets, i.e., the sample size is 11 × 30 = 330 for each contrast at the method level and 30 at the individual dataset level.

Next, we analyze the performance for each discretization technique in terms of the average rank achieved for the various datasets. This is done using the Friedman and Holm tests, which have been widely considered in data science for comparing the performance of different methods and assessing statistical significance [60]. The AUC and KS metrics are considered for this analysis.

The Friedman test with Iman-Davenport correction evaluates the null hypothesis of equal ranks for all discretization techniques. The chi squared statistics obtained for the this test were 27.51 and 48.69 for the AUC and KS measures, respectively, rejecting the null hypothesis in both cases with a *p* value below 0.001. We can conclude that some discretization strategies are able to statistically outperform others.

The Holm post hoc test is used to perform pairwise comparisons between the discretization technique with the highest average rank and those remaining. For a given technique that achieved the *j*-th position in the rank, each pairwise test is rejected when the *p* value is below a significance threshold *α*/(*j* − 1), with *α* = 5% and *j* = 2, …, 6. The results for the Holm post hoc test are provided in Tables 2 and 3 for the AUC and KS measures, respectively. For each discretization technique, these tables also include the average rank and the average value for each metric.

**Table 2:** Holm post hoc test for AUC.

Method	Rank	AUC	*p* value	*α*/(*j* − 1)	Outcome
DCC	1.636	0.769	-	-	-
DEC	1.818	0.769	0.820	0.050	not reject
QUA	3.182	0.755	0.053	0.025	not reject
GAU	3.909	0.635	0.004	0.017	reject
KME	4.864	0.575	0.000	0.013	reject
UNI	5.591	0.530	0.000	0.010	reject

AUC as performance metric for the various discretization strategies.

**Table 3:** Holm post hoc test for KS.

Method	Rank	KS	*p* value	*α*/(*j* − 1)	Outcome
DCC	1.545	0.389	-	-	-
DEC	1.727	0.388	0.820	0.050	not reject
QUA	3.091	0.365	0.053	0.025	not reject
GAU	4.000	0.183	0.002	0.017	reject
KME	4.864	0.118	0.000	0.013	reject
UNI	5.773	0.038	0.000	0.010	reject

KS as performance metric for the various discretization strategies.

It can be observed in Tables 2 and 3 that the test achieves consistent results for the two metrics. Results show that DCC, DEC, and QUA can be considered statistically equivalent given the null hypothesis rejection of the Holm test and, simultaneously, statistically different from their counterparts GAU, KME, and UNI.

Analyzing in detail, the method with the best average performance is DCC, statistically outperforming GAU, KME, and UNI. On the other hand, the DEC method achieves very similar performance compared to DCC, concluding that these two discretization techniques generally lead to the best results, even if they perform closely to QUA; additional qualitative gains for not restricting the segments to equal frequency can be achieved by using DCC and DEC instead of QUA.

The statistical evidence demonstrates that using a combined discretization strategy based on clustering is a feasible alternative and maintains the model’s interpretability and competitiveness for its classification quality.

As for the computational cost (indicated in Table 4), the DEC combination naturally has a higher cost due to the method’s comprehensiveness. However, the approximate four-second average has not been deemed a limitation for its implementation. Additionally, the DCC combination does not have significantly different computational times compared with its univariate counterparts.

**Table 4:** Computational time.

Method	Mean	95% CI
DCC	1.1319	[0.9224, 1.3414]
DEC	4.1383	[3.3257, 4.9508]
EQD	1.1066	[0.8968, 1.3165]
EQF	1.1469	[0.935, 1.3588]
GMM	1.1777	[0.9602, 1.3951]
KME	1.1777	[0.9614, 1.3939]

Seconds per fold comparative chart.

## 7 Conclusions and future work

This paper proposed a combination of discretization methods that improve the classification quality of logistic regression as a binary classifier. The combination of discretization methods was conducted using the continuous predictor variables available on each dataset. Subsequently, performance metrics of the proposed combination were obtained in eleven different and diverse datasets through computation experimentation. Experimental results showed that the proposed method has successfully achieved the objectives pursued. Firstly, an alternative discretization through Gaussian mixture models and *k* means proved its competitiveness by maintaining an appropriate classification quality and supplementing widely used conventional discretization methods. Furthermore, combining discretization methods increases classification quality compared to individual methods when applied in various credit risk datasets. On the other hand, since the created discrete segments are univariate, the classifier’s explainability is not affected.

The proposed combination method could potentially fill a void in the fields of application where explainability is crucial for a model based on statistical learning simultaneously with permanent efforts to improve classification quality. Similarly, the proposed method provides a balance between modern assembly techniques and the simplicity of linear models, thus benefiting from both approaches’ advantages.

Future work concerns assembling discretization methods through genetic algorithms to determine the optimal combination of clusters and methods to be included in the final selection since the search is performed thoroughly and less efficiently in this stage. Alternatively, increasing the number of discretization techniques is plausible, provided that the original explainability objectives are preserved. Findings suggest the proposed method can be successfully incorporated as a discretization alternative in the development of credit scoring models without a significant impact on operation, explainability, and implementation and with a significant contribution to better performance of a logistic regression model used in a classification problem. Finally, the applications of the proposed method scale to every industry where a binary classification problem arises. Examples such as predictive maintenance and predictive quality in manufacturing, customer analytics in retail, or disease detection in healthcare are a sample of compelling use cases worth being explored as an extension of the present work.
